# Stereoscopic depth constancy

**DOI:** 10.1098/rstb.2015.0253

**Published:** 2016-06-19

**Authors:** Phillip Guan, Martin S. Banks

**Affiliations:** 1 UC Berkeley-UCSF Graduate Program in Bioengineering, Berkeley and San Francisco, CA 94720, USA; 2 School of Optometry, Vision Science Program, UC Berkeley, Berkeley, CA 94720, USA

**Keywords:** stereopsis, depth perception, human vision

## Abstract

Depth constancy is the ability to perceive a fixed depth interval in the world as constant despite changes in viewing distance and the spatial scale of depth variation. It is well known that the spatial frequency of depth variation has a large effect on threshold. In the first experiment, we determined that the visual system compensates for this differential sensitivity when the change in disparity is suprathreshold, thereby attaining constancy similar to contrast constancy in the luminance domain. In a second experiment, we examined the ability to perceive constant depth when the spatial frequency and viewing distance both changed. To attain constancy in this situation, the visual system has to estimate distance. We investigated this ability when vergence, accommodation and vertical disparity are all presented accurately and therefore provided veridical information about viewing distance. We found that constancy is nearly complete across changes in viewing distance. Depth constancy is most complete when the scale of the depth relief is constant in the world rather than when it is constant in angular units at the retina. These results bear on the efficacy of algorithms for creating stereo content.

This article is part of the themed issue ‘Vision in our three-dimensional world’.

## 1. Introduction

The retinal image of an object varies depending on the overall illumination of the object, the colour of the illuminant, the orientation of the object relative to the illuminant, the orientation and distance of the viewer relative to the object and more. Yet we will perceive this object to be relatively constant in brightness, colour, size and shape across many different viewing conditions and environments. These constancies are a crowning achievement of biological vision in that they enable relatively invariant percepts despite varying inputs. Here we focus on constancies associated with depth perception: specifically, the ability to perceive constant depth even when the spatial scale of depth variation changes and the ability to see constant depth when viewing distance changes.

An understanding of how the visual system estimates depth from retinal images has become increasingly important with the emergence of virtual- and augmented-reality systems that aim to create realistic three-dimensional experiences. There are many depth cues that can be used to achieve this goal, and they can be broadly classified into triangulation cues (stereo, motion, focus), perspective cues (linear perspective, texture gradients, relative size) and light-transport cues (occlusion, shading, aerial perspective). From a geometrical perspective, some of these cues provide only ordinal information (e.g. occlusion); that is, they indicate only the distance ordering of objects in the environment. Other cues provide only relative distance information (e.g. linear perspective); that is, they indicate ratios of distances, but cannot by themselves indicate metric distances. Other depth cues, including all triangulation cues and one perspective cue (familiar size) can provide metric distance information; that is, how far objects are from the viewer. Here we concentrate on stereoscopic cues, because the signals required to convert retinal signals (binocular disparity) into distance estimates are known and, because stereopsis is probably the most precise of metric distance cues under ordinary viewing conditions.

When a stimulus with fixed relief is viewed at two different viewing distances, two aspects of the retinal images change. (i) The spatial scale of the relief shrinks at the retina in proportion to the increase in distance. (ii) The amplitude of the variation in disparity decreases in proportion to the distance squared. Thus, to perceive the relief as constant—i.e. to achieve depth constancy—the visual system must compensate for changes in spatial frequency and for changes in disparity amplitude. In the first experiment, we examined how depth is perceived when spatial frequency changes, but viewing distance remains the same. We found that subjects perceive the same amount of depth from disparity across spatial frequency as long as the disparity amplitude is sufficiently above threshold. Because there was only one viewing distance, depth constancy and disparity matching would lead to the same results. In the second experiment, we repeated the first one, but placed the stimuli at different distances, so we could differentiate between depth constancy and disparity matching. All cues to distance—vergence, vertical disparity and focus cues—were veridical. We found that depth constancy, and not disparity matching, is observed across a broad range of spatial frequencies.

## 2. Experiment 1: depth constancy across spatial frequency

### (a) The problem

**Figure 1.**
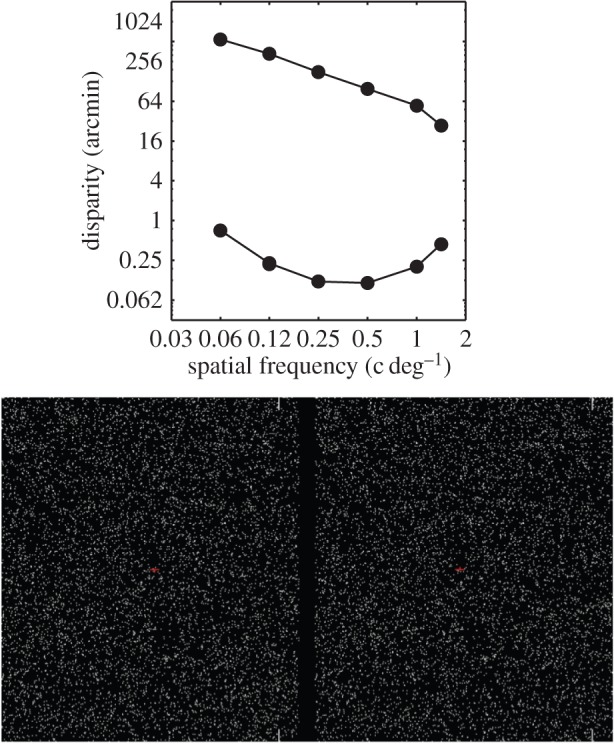
The limits of visibility for depth from disparity. The top panel shows the lower-disparity thresholds (minimal disparity required to perceive depth) and the upper-disparity limits (maximum disparity that supports depth perception) as a function of spatial frequency (adapted from Kane *et al.* [1]). The bottom panels demonstrate those limits (adapted from Kane *et al.* [1]). Cross fuse by directing the left eye to the right panel and the right eye to the left panel. Disparity increases from left to the right and spatial frequency increases from bottom to top. The smallest visible disparity varies with spatial frequency with low and high frequencies requiring more disparity than medium frequencies. The largest visible disparity also varies with spatial frequency with the largest value decreasing monotonically with frequency. (Online version in colour.)

To achieve depth constancy, the visual system must compensate for changes in the spatial frequency of the depth relief. Constancy occurs if a given depth interval is perceived as having the same depth even when the depth variation occurs at different spatial scales. Figure 1 shows how the ability to perceive depth from disparity is affected by the spatial frequency of disparity variation. The smallest detectable disparity occurs at a frequency of approximately 0.3 cpd; at lower or higher frequencies, disparity variation must be significantly larger to be perceived [1,2]. In addition, there is a largest disparity that produces a coherent depth percept and that disparity is strongly dependent on spatial frequency [1,3]. These limits are by-products of the computations made to estimate disparity [1,4–9], and thus one wonders if the visual system is able to take the lower-disparity thresholds and upper-disparity limits into account in a way that enables depth constancy.

Similar issues arise in the luminance domain; the ability to detect luminance variation is strongly dependent on spatial frequency [10]. The cause of the variation in contrast threshold is mostly the low-pass filtering of the eye's optics [11–13]. Georgeson & Sullivan [14] examined how such differences in threshold affect the apparent contrast of suprathreshold luminance gratings. They presented two gratings that had rather different contrast thresholds. When the gratings were above threshold, observers judged the contrasts of the two gratings to be the same when they were physically the same. Because of the low-pass filtering of the eye's optics, this means that observers perceived gratings to have the same contrast even when their retinal-image contrasts differed substantially. Georgeson and Sullivan called this phenomenon *contrast constancy*. In the first experiment, we asked whether an analogous constancy occurs across spatial frequencies of disparity variation. The comparison between contrast and depth constancy is interesting and similar behaviour across the two domains is not guaranteed, because the parts of the visual process that create the need to compensate differ in the two phenomena. For contrast constancy, the differences in threshold across spatial frequency are primarily caused by optical filtering before the stimulus lands on the retina. For stereoscopic depth constancy, the differences in disparity threshold as a function of spatial frequency have a neural, not an optical, basis [1].

### (b) Methods

#### (i) Observers

Four people (two males and two females) 22–31 years of age participated. One was an author. The others were unaware of the experimental hypotheses. All had normal stereoacuity as measured by a Randot stereo test. Interpupillary distance (IPD) was measured using a ruler in a standard clinical procedure.

#### (ii) Apparatus

The stimuli were presented on a mirror stereoscope with two cathode ray tube (CRT) displays (Iiyama HM204DT). The lines of sight from the two eyes were reflected from mirrors near the eyes such that the lines were collinear with normals from the centres of the display screens. The experiment was conducted in a dark room, so the displays provided the only visible light input to the eyes. Each display's resolution was 800 × 600 pixels. At the 115 cm viewing distance, pixels subtended 1.5 arcmin. By using anti-aliasing, we could present much smaller disparities than that. The smallest disparity we presented was 15 arcsec. Vergence distance was the same as the optical distance from eye to screen. Refresh rate was 200 Hz.

#### (iii) Stimuli and procedure

Identical dynamic random-dot patterns were presented to the two eyes between trials. A fixation target composed of two binocular horizontal line segments and two dichoptic vertical segments was also presented at all times. Observers monitored the apparent alignment of the dichoptic segments to make sure that fixation was accurate before initiating a stimulus presentation. They were also told to maintain fixation on the fixation target during the stimulus presentation.

**Figure 2.**
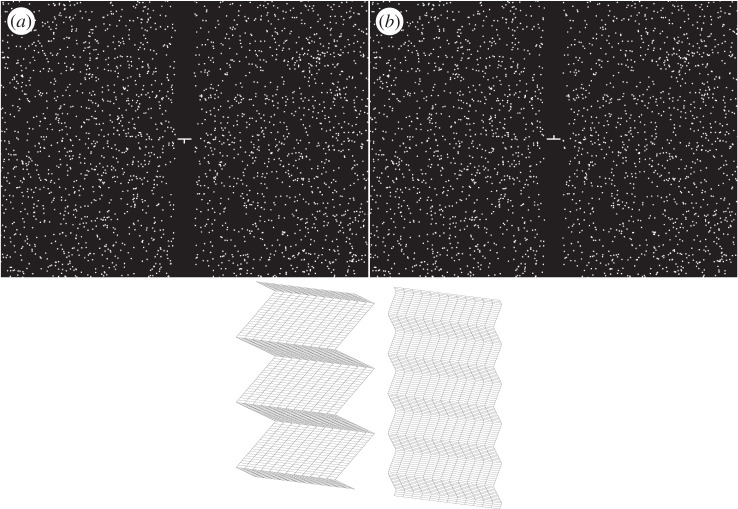
(*a*,*b*) Example stimuli. The standard stimulus is on the left and the comparison on the right. The top panels can be cross-fused to see the depth corrugation. The bottom panel indicates the depth variation being presented.

Two stimuli generated using PsychToolbox [15,16] were presented on every trial: a standard and a comparison. They were both random-dot stereograms depicting triangular-wave corrugations in depth and were each 15° tall and 9° wide. Figure 2 provides an example. Because the corrugations were horizontal, the left- and right-eye images differed in row-by-row displacements of the dots, yielding no monocular density cues. The inner edges of the patches were 0.5° from the centre of the fixation target. The dots in the stereograms were 3 arcmin in diameter. Dot density was 9 dots deg^−2^, yielding a Nyquist frequency of 1.5 cpd for each frame [8]. However, new dots consistent with the simulated waveform were presented every 5 msec, so the effective dot density (and therefore the effective Nyquist frequency) was much higher [17]. There were unmatched dots (i.e. seen by one eye but not the other) near the left and right edges of the stimuli; the number of such dots increased with disparity amplitude. However, the unmatched dots did not hinder task performance, because the central portion with matched dots was large enough to compare apparent depths easily.

The positions of the standard and comparison stimuli (left or right) varied randomly from trial to trial. After each presentation, observers indicated with a button press the corrugation (left or right) that had greater apparent variation in depth. The spatial frequency of the standard stimulus was always 0.3 cpd. It had one of eight peak-to-trough disparities: 0.25, 0.5, 1, 4, 16, 32, 64 or 96 arcmin. The comparison stimulus had one of eight spatial frequencies: 0.0625, 0.125, 0.25, 0.5, 0.75, 1, 1.5 or 2 cpd. Its disparity was varied trial by trial according to the method of constant stimuli. The various combinations of standard and comparison stimuli were randomly interleaved within a session. The experiment yielded 64 psychometric functions for each observer, one for each pairing of standard disparity and comparison disparity and spatial frequency.

#### (iv) Data analysis

Responses ranged from 0% to 100%, where 0% corresponded to disparities for which the comparison stimulus always appeared to have less depth than the standard, and 100% corresponded to disparities for which the comparison always appeared to have more depth. Each set of psychometric data was fit with a cumulative Gaussian using a maximum-likelihood criterion [18]. The 50% point on the fitted function was the estimate of the disparity of the comparison stimulus that appeared on average to have the same depth as the standard.

The disparity gradient—the change in disparity with change in position in the frontal plane—places an upper bound on the largest disparity that supports depth perception [3]. Specifically, when the change in disparity is roughly the same as the change in position (a gradient of approx. 1), the perception of depth from disparity collapses [1,3]. The disparity-gradient limit affected our data, because as the disparity of the comparison was increased to match the apparent depth of the standard, the gradient limit was sometimes exceeded resulting in a loss of perceived depth altogether. The psychometric data in such cases were non-monotonic and could not be fit with a cumulative Gaussian. To deal with this, we discarded the data from conditions in which the response (i.e. comparison judged to have more depth than standard) did not exceed 85%. Similarly, when the disparity of the standard was near the disparity threshold, the standard and comparison stimuli both appeared flat when the comparison had a smaller disparity than the standard, so observers responded randomly. To address this, we discarded data from conditions in which the response never fell below 15%.

Three observers provided approximately 9000 responses and one provided approximately 11 000. The results were quite similar for all observers, so we pooled the data across observers and fit the resultant with a cumulative Gaussian.

### (c) Results

**Figure 3.**
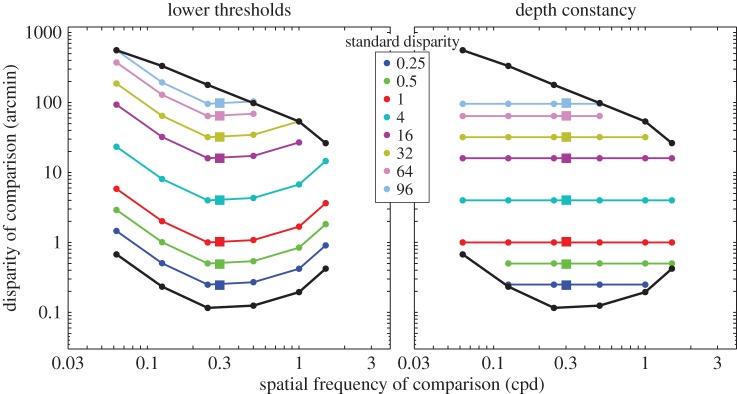
Possible outcomes for experiment 1. Each panel plots disparity of the comparison stimulus as a function of spatial frequency of the comparison stimulus. The standard stimulus has a fixed spatial frequency of 0.3 cpd. It is represented in both panels by the coloured squares. The lower-disparity thresholds and upper-disparity limits from Kane *et al*. [1] are represented by the black contours and points. The left panel shows the expected data if the disparity required to make the comparison stimulus have the same perceived contrast as the standard stimulus follows the variation in the lower thresholds as a function of spatial frequency. The comparison stimulus would always require more disparity than the standard for it to appear to have the same depth variation. Those predictions are represented by the coloured lines and circles. The right panel shows the expected data if depth constancy occurs. In this case, the observer would perceive the same depth in the comparison and standard stimulus when they had the same physical disparity. Again, the predictions are represented by the coloured lines and circles.

In this experiment, we measured how perceived depth from disparity is affected by the spatial frequency of depth variation. Possible outcomes are depicted in figure 3. The black curves and points represent the lower-disparity thresholds and upper-disparity limits from Kane *et al*. [1]. The left panel shows the outcome if the variation in perceived depth across spatial frequency has the same form as the lower-disparity thresholds. In this case, low- or high-frequency corrugations would always require more disparity than a corrugation of 0.3 cpd for them to appear to have the same depth variation. Quantitatively, if the ratio of lower-disparity thresholds—threshold of the comparison at spatial frequency *f* divided by the threshold of the standard at 0.3 cpd—is *R*, then the ratio of disparities for equal perceived depth would be *R* for all disparities of the standard. This outcome would occur if the processes responsible for different thresholds at different spatial frequencies affected perceived depth in the same fashion as might be expected for computational and neural models of disparity estimation [4,5,8].

The right panel shows the outcome if depth constancy occurs: that is, if observers perceive the same depth variation in two stimuli when they have the same physical disparity. Here the isodepth contours are horizontal lines.

**Figure 4.**
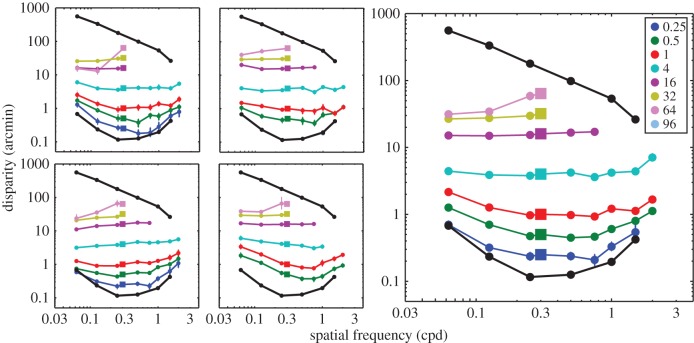
Perceived depth for various corrugation frequencies. The disparity of the comparison stimulus that matched the perceived depth of the standard stimulus is plotted as a function of the spatial frequency of the comparison stimulus. The left panels show the data from individual subjects. The right panel shows the data pooled across subjects. The standard stimulus had a spatial frequency of 0.3 cpd. It had one of eight disparities as indicated by the coloured squares. The disparity of the comparison that matched the perceived depth of the standard is indicated by the coloured circles. Error bars represent 95% confidence intervals.

Figure 4 shows the results for each observer and for the data pooled across observers. When the disparity of the standard was near threshold, the data were similar to the lower-disparity thresholds, i.e. similar to the left panel of figure 4. However, when the disparity of the standard was larger, the data became flat; this behaviour is consistent with depth constancy as in the right panel of figure 4. When the disparity of the standard was yet larger, it approached the disparity-gradient limit. In that case, we could not obtain reliable depth matches for comparison frequencies greater than 0.3 cpd, because those stimuli exceeded the gradient limit and yielded no perceived depth. No data are plotted for those conditions.

The data for relatively small disparities appear to be similar to data reported, but not published, by Ioannou *et al.* [19]. They observed depth constancy for the range of disparities they tested (0.5–8 arcmin) (fig. 19.24d; [20]).

**Figure 5.**
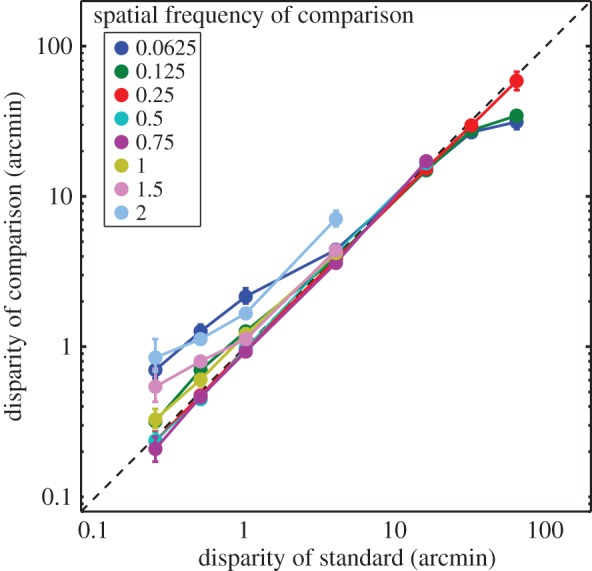
The comparison stimulus that produced the same perceived depth as the standard stimulus. The matching disparity of the comparison for the pooled data is plotted as a function of the disparity of the standard. The dashed line represents depth constancy: where the data would lie if observers perceived the same depth when the disparities were the same. The colours represent the data for different comparison spatial frequencies. Error bars are 95% confidence intervals.

Figure 5 plots the data from figure 4 in a different way. It shows the disparity of the comparison stimulus required to match the perceived depth of the standard stimulus for different disparity amplitudes. The dashed diagonal line represents depth constancy: in this case, perceived depth of the standard and comparison stimuli should be the same when their physical disparities are the same. If the data were consistent with the lower-disparity thresholds (left panel, figure 3), then they would fall on diagonal lines above the dashed line; there would be a different diagonal line for each spatial frequency. The data are generally very consistent with the constancy prediction. They deviate in only two cases: (i) When the disparity of the standard is near threshold: in this case, the data are above the constancy line, meaning that subjects needed more disparity in the comparison stimulus than in the standard for them to have equal perceived depth. This behaviour is similar to the lower-disparity thresholds (left panel, figure 3). (ii) When the disparity of the standard is high such that it approaches the disparity-gradient limit: here the data fall below the constancy line.

**Figure 6.**
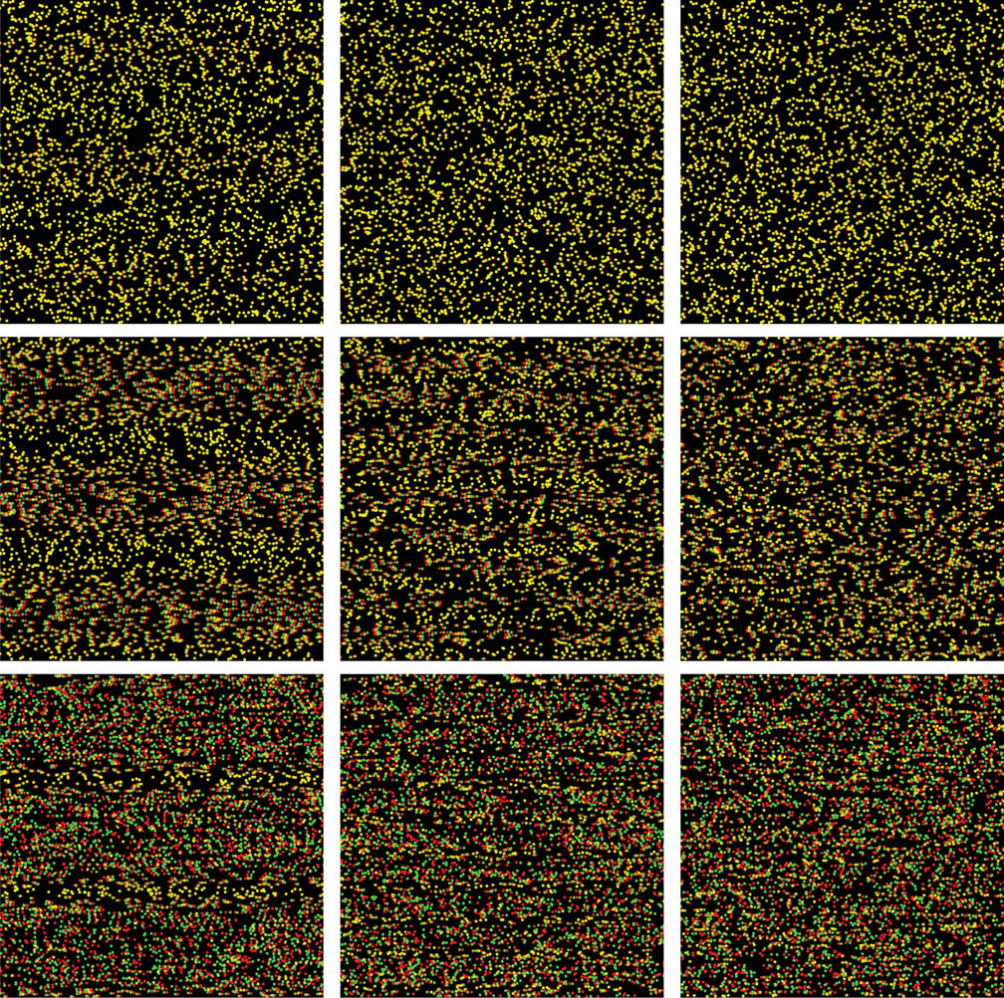
Perceived depth for different disparities and spatial frequencies. View from 60 cm with red/green anaglyph glasses. The spatial frequency in each column and the disparity in each row is the same. Spatial frequency increases from left to right and disparity increases from top to bottom. Most observers report that the apparent depth in the top row varies with spatial frequency even though the disparities are the same. Most report that the apparent depth in the middle row does not vary with spatial frequency, i.e. depth constancy occurs. Most observers report that the apparent depth in the bottom row varies with spatial frequency, because the disparity-gradient limit is exceeded in the right panel.

Figure 6 provides a demonstration of the main findings in experiment 1. Each column of panels has one spatial frequency, and that frequency increases from left to right. Each row has one disparity, which increases from top to bottom. The disparity in the top row is close to the threshold for detecting disparity. In that row, the apparent depth variation of the left and right panels is less than that of the middle panel. Here, depth constancy is not observed, similar to the data at small disparities in figures 4 and 5. The disparity in the middle row is well above threshold, and the three stimuli appear to have the same depth variation; i.e. depth constancy is observed, similar to suprathreshold disparities in figures 4 and 5. The disparity in the bottom row is large, and for higher spatial frequencies, it approaches or exceeds the disparity-gradient limit. In that row, the apparent depth of the right panel is much less than that of the other panels because it has exceeded the gradient limit.

Despite the many differences in the neural processing of disparity and luminance, our data on depth constancy in the disparity domain are quite similar to the data on contrast constancy in the luminance domain. Specifically, constancy is observed in both domains when the stimuli are above threshold and is not observed when they are at or near threshold. The one clear difference between depth constancy and contrast constancy concerns the perception of depth when the stimulus approaches the disparity-gradient limit. The limit places an upper bound on the disparities at which depth can be perceived: a phenomenon that has no analogue in the luminance domain.

**Figure 7.**
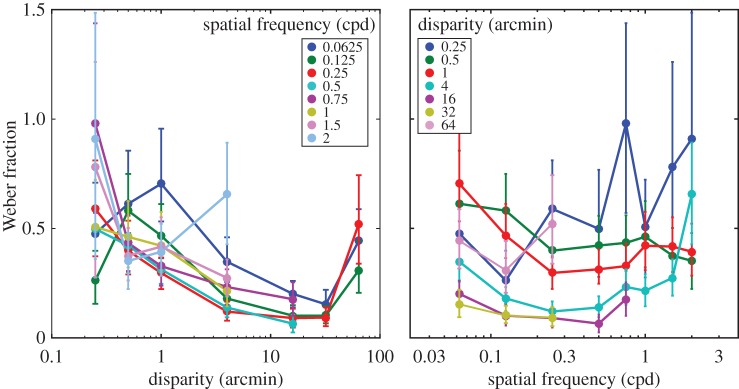
Just-notable differences (JNDs) in experiment 1. In the left panel, the abscissa represents the standard disparity and the ordinate JNDs. The ordinate values are Weber fractions (the numerator is the base disparity minus the average of the just-notable increment and decrement and the denominator is the base disparity). Different colours represent different spatial frequencies (see inset). In the right panel, the abscissa represents spatial frequency and the ordinate again JNDs. Different colours represent different disparities (see inset).

We also examined just-noticeable differences (JNDs) for the various conditions of the experiment. Figure 7 shows those JNDs plotted in two ways: one as a function of disparity and the other as a function of spatial frequency. Kane *et al*. [1] reported that JNDs at the lower-disparity threshold were larger than JNDs at the upper-disparity limit. Our results are consistent with that. In addition, we found that JNDs generally decreased as disparity increased, but increased again at the largest disparity. The increase in JNDs at the largest disparity is due to inconsistently perceiving depth from the standard as it approached the upper-disparity limit. The effect of spatial frequency on JNDs is relatively small except when higher frequencies approached the disparity-gradient limit causing an increase in JNDs.

In conclusion, the visual system is able to compensate for spatial-frequency-dependent differences in threshold, provided that the disparity variation is suprathreshold. Thus, the system achieves the ability to perceive a fixed interval in depth as constant even when the spatial scale of the depth change varies. This behaviour could be due to matching disparity magnitudes (disparity matching) or to actually perceiving the same depth across scale (depth constancy). Experiment 2 was designed to determine which of two strategies provides a better account for the behaviour of the visual system.

## 3. Experiment 2: depth constancy across spatial frequency and viewing distance

### (a) The problem

**Figure 8.**
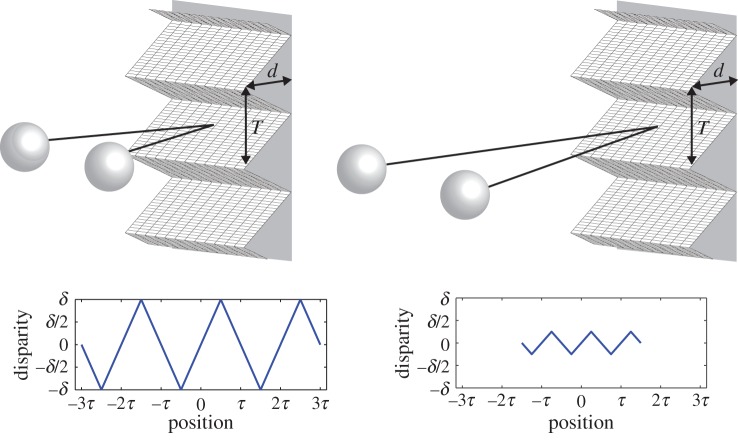
The change in retinal disparity and spatial frequency with a change in viewing distance. *d* and *T* represent, respectively, the peak-to-trough depth and the period of a corrugation in the real world. *δ* and *τ* represent the disparity and period of the corrugation on the retina at the shorter viewing distance. Doubling the viewing distance reduces disparity from *δ* to *δ/*4 and reduces the period from 2*τ* to *τ*. (Online version in colour.)

**Figure 9.**
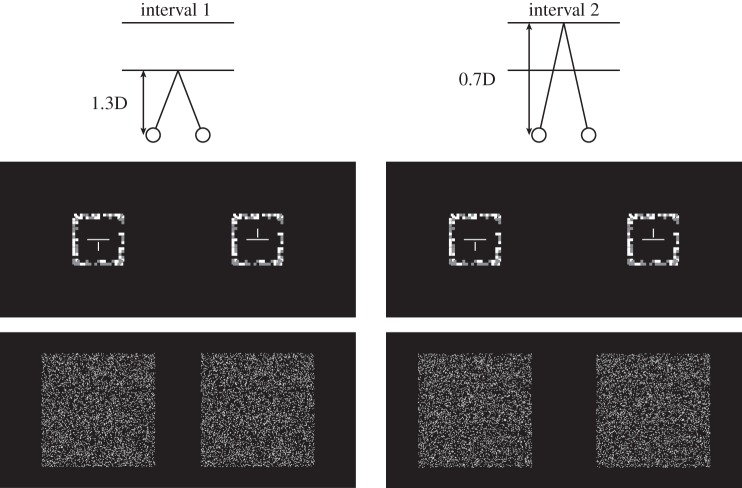
Stimuli in experiment 2. The left side shows an example of the fixation target and stimulus in interval 1 and the right side shows an example in interval 2. The fixation target shown in the middle row had a binocular horizontal line segment and dichoptic vertical segments. This allowed subjects to achieve accurate vergence and accommodation for the upcoming stimulus presentation. Examples of the stimuli are shown in the bottom row. They were triangular-wave corrugations in depth presented at two different distances. They were shown in temporal sequence, not simultaneously as suggested here.

When the distance to a depth-varying object is increased, the spatial frequency of the depth variation increases in proportion to distance while the disparity of the variation decreases in proportion to the distance squared. These two effects are illustrated in figure 8. In other words, the same object generates different magnitudes of horizontal disparity depending on viewing distance. Because of this, the metric structure of the environment cannot be estimated from horizontal disparity alone. We examined the combined effects of viewing distance and spatial frequency on depth constancy by repeating experiment 1 but with the standard and comparison at different distances (figure 9). To achieve depth constancy, the visual system must estimate the distances to the two stimuli and use those estimates to scale perceived depth from disparity at the retina. There are three signals the visual system could use to estimate viewing distance in our viewing situation: vergence, vertical disparity and accommodation [21–23]. By vergence, we mean the vergence response of the eyes and extra-retinal signals used to measure it. By vertical disparity, we mean the horizontal and vertical gradients of vertical disparity, which tend towards zero with increasing distance. By accommodation, we mean the accommodative response of the eyes and extra-retinal signals used to measure that response.

Some previous studies of stereoscopic depth constancy have manipulated vergence and vertical-disparity signals and most showed that both signals are in fact used [21,22,24–26]. To the best of our knowledge, only one has presented appropriate accommodation signals and it showed that there was indeed an effect of the accommodative stimulus [23]. None examined the effect of the spatial frequency of depth variation on depth constancy in combination with the effect of viewing distance.

We wanted to manipulate vergence, vertical disparity and accommodation together, so that we could determine whether the visual system achieves depth constancy when those signals all specify the same distance and vary appropriately for stimuli at different distances. To accomplish this, we used a stereoscopic multi-plane display [27] that allowed us to manipulate all three cues appropriately.

### (b) Methods

#### (i) Observers

Four people participated, not all the same ones as in experiment 1. One was male and three were female. Their ages were 20–27 years. One was an author, and the others were naive to the purpose of the experiment. All had normal stereoacuity as measured by a Randot stereo test. IPD was measured with a ruler in a standard clinical procedure.

#### (ii) Apparatus

Details concerning the stereoscopic multi-plane display are provided in Love *et al*. [27]. It consists of two CRTs, one for each eye and high-speed, switchable lenses that allow rapid change in focal distance synchronized with the display of different parts of the simulated three-dimensional scene. The stimuli delivered to each eye are presented on four presentation planes, separated by approximately 0.6 dioptres (D). The accommodative stimuli at those four distances are precise. With depth-weighted blending [28], we can also create a reasonable approximation of accommodative stimuli in-between the planes [29,30]. However, in the experiments reported here, we did not require such blending, because the stimuli were always presented on one of the presentation planes. The vergence and accommodation distances were always equal to one another and were either 77 (1.3D) or 143 cm (0.7D). We calibrated the display so that when the vergence distance was 77 cm (1.3D) the central surface normals from the CRTs were collinear with the visual axes of the observer's eyes when the observer was fixating the centres of the screens.

#### (iii) Stimulus and procedure

The stimuli were again random-dot stereograms depicting triangular-wave corrugations in depth. The standard stimulus had a peak-to-trough depth of 1, 4 or 16 cm in world coordinates. The comparison stimulus was presented at a different distance. For half the trials, the standard was rendered and displayed at 0.7D and the comparison at 1.3D, and for the other half the standard was rendered and displayed at 1.3D and the comparison at 0.7D. We varied the peak-to-trough depth of the comparison by varying its disparity until its depth in the world appeared to be equal to that of the standard.

The spatial frequency of the standard at the retina was always 0.3 cpd. This corresponds to 0.12 cycles cm^−1^ when the standard is presented at 0.7D and 0.22 cycles cm^−1^ when presented at 1.3D. The frequency of the comparison at the retina was 0.125, 0.3, 0.5 or 1 cpd (0.09, 0.22, 0.37 and 0.74 cycles cm^−1^ when presented at 1.3D, and 0.05, 0.12, 0.20 and 0.40 cycles cm^−1^ when presented at 0.7D). There were 24 conditions in all. The peak-to-trough depths and spatial frequencies were chosen to cover the range of behaviours observed in experiment 1: small disparities near the lower-disparity threshold, suprathreshold disparities for which depth constancy was observed and large disparities near the disparity-gradient limit.

To allow observers to view stimuli at different distances, we presented them in two successive intervals in random order: one stimulus at 0.7D and the other at 1.3D. Before the presentation of the stimulus in an interval, a fine binocular and dichoptic cross was presented (top panel, figure 9) and subjects adjusted their vergence to subjectively align the limbs of the cross (thereby assuring accurate vergence) and adjusted their accommodation to sharpen the cross (assuring accurate accommodation). After they were satisfied with the alignment and sharpness, they initiated a 1 s stimulus presentation with a button press. The stimulus appeared at the same vergence and accommodative distance as the fixation cross. Then, the cross would re-appear at the other distance, and the subject would adjust vergence and accommodation to that distance and initiate the stimulus presentation in the second interval with a button press. The stimulus of course appeared at the same vergence and accommodative distance as the fixation cross. After both intervals had been presented, subjects indicated the one in which the triangular-wave corrugation appeared to have greater peak-to-trough depth in the world. We used the method of constant stimuli to vary the disparity of the comparison stimulus to find the point at which subjects picked the comparison and standard stimuli equally often. A total of 100–150 trials were completed for each subject in each condition (excluding the conditions near the disparity-gradient limit) resulting in approximately 2500 trials per subject. All conditions were randomly interleaved.

#### (iv) Rendering stimuli

We used perspective projection to ensure that the vertical disparities in the stereograms were correct for the intended viewing distances. This rendering method introduces perspective effects into our stimuli that were not present in experiment 1. The potential impact of these effects is discussed in the Results. We first defined the *xyz* world coordinates of a triangular-wave corrugation in three-dimensional space; the phases of the standard and comparison stimuli were randomly given values from 0° to 180° in increments of 30°. We calculated the left- and right-eye perspective projections for 7500 points over a 20° × 20° area (18.75 dots deg^−2^, a Nyquist frequency of 2.17 cpd; [8]). We used each observer's IPD in generating the stimuli, which means that the disparity presented to each observer differed according to their IPD. After performing the projection, the left and right edges of the stereogram were notably corrugated. That corrugation could have been used as a monocular cue to depth. We eliminated this possibility by cropping the stimulus such that only the central 17 × 17° portion was visible (figure 9).

We wanted to make sure that the distribution of dots in the monocular images was uniform to eliminate variations in dot density as a monocular cue to depth. To accomplish this, we created a uniform random distribution of dots on a frontal plane at 0.7 or 1.3D and projected those dots onto the surface of the triangular-wave corrugations from the perspective of the cyclopean eye using back projection [31]. We then calculated the perspective projections of the dots for the left and right eyes. This procedure yielded a dot distribution that appeared uniform in the monocular images.

The display was set up such that the screens were perpendicular to the lines of sight when the eyes were converged at 1.3D. To accurately present stimuli at 0.7D, we rotated and translated the stimulus virtually to ensure that the surface normal of a simulated frontal plane was collinear with the visual axes of the eyes when converged at 0.7D. We changed vergence distance by translating the left- and right-eye images to create the correct vergence angle for each subject. Finally, we changed accommodative distance by changing the plane on which the stimuli were presented in the multi-plane display.

#### (v) Data analysis

The methods for fitting and evaluating the psychometric data were the same as before. All conversions from world coordinates to disparity were done for an IPD of 6.1 cm, which is the average value for the four observers. Their actual values were 5.9, 6.1, 6.1 and 6.3 cm.

### (c) Results

**Figure 10.**
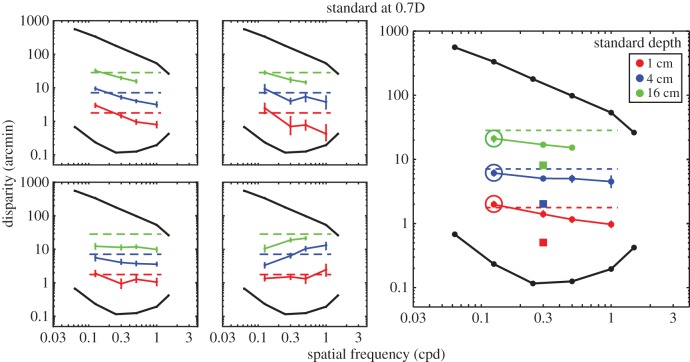
Perceived depth from disparity across spatial frequency and viewing distance. The standard was presented at 0.7D and the comparison at 1.3D. The panels on the left show the individual subject data, and the one on the right shows data once pooled across subjects. Each panel plots the disparity of the comparison (circles) that yielded the same perceived depth as the standard (squares). Different colours represent matches for different peak-to-trough depths of the standard. The dashed horizontal lines represent the disparities that would yield the same perceived depth if depth constancy were observed. The three larger circles indicate the comparison stimuli that have approximately the same spatial frequency in the world as the standard stimulus. The black lines and circles represent the lower-disparity thresholds and upper-disparity limits from Kane *et al*. [1]. Error bars represent 95% confidence intervals.

**Figure 11.**
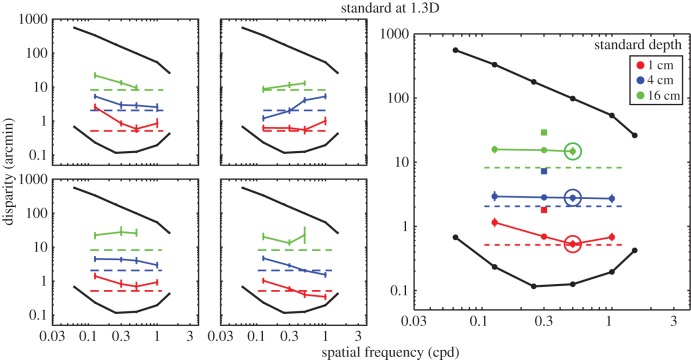
Perceived depth from disparity across spatial frequency and viewing distance. The standard was presented at 1.3D and comparison at 0.7D. Left and right panels show the individual subject data and pooled data, respectively. Each panel plots the comparison disparity (circles) that yielded the same perceived depth as the standard (squares). Different colours represent matches for different peak-to-trough depths of the standard. Dashed horizontal lines represent disparities that would yield the same perceived depth if depth constancy were observed. The larger circles indicate the comparison stimuli that have approximately the same spatial frequency in the world as the standard. Error bars are 95% confidence intervals.

We examined how changes in viewing distance and spatial frequency at the retina are taken into account. Figures 10 and 11 show predictions and the results. The standard stimulus always had a spatial frequency of 0.3 cpd and had one of three peak-to-trough depths in the world (1, 4 or 16 cm). The comparison stimulus had different spatial frequencies and was always presented at a different distance than the standard.

In experiment 1, we demonstrated that the visual system compensates for the effects of spatial frequency to achieve depth constancy. In that experiment, however, the standard and comparison were presented at the same viewing distance, so the predictions for depth constancy and disparity matching (setting the peak-to-trough disparities to the same value) were the same. By putting the standard and comparison at different distances, these two predictions are dissociated. In experiment 2, if subjects failed to take distance into account and simply matched the disparities at the retina, then the data would fall on horizontal lines through the squares, which represent the disparities of the standard (disparity matching). Those lines are not shown in the figures, but such data would be a failure of depth constancy. If, on the other hand, observers saw the same depth variation when the two stimuli specified the same peak-to-trough depth in the world, then they would require more disparity in the comparison stimulus than in the standard when the comparison was nearer than the standard, and they would require less disparity in the comparison than the standard when the comparison was farther than the standard. The dashed lines in the two figures represent those predictions. In figure 10, the comparison was presented at 1.3D and the standard at 0.7D, so more disparity would be needed in the comparison than in the standard to achieve depth constancy. In figure 11, the situation is reversed, so less disparity would be required in the comparison to achieve constancy. The results from experiment 2 are represented by the coloured lines and circles.

The results show that people do take viewing distance into account and thereby perceive roughly the same depths in stimuli that have the same physical depth but are viewed from different distances. Recall that disparity matching would yield data that fell on horizontal lines through the squares. In each case, the data are much closer to the depth–constancy predictions represented by the horizontal dashed lines. However, there were fairly consistent deviations from complete constancy. When the comparison was closer than the standard (figure 10), the deviations were downward in the figure, meaning that subjects required less disparity in the comparison with match the standard than would be predicted if they were perfectly constant. When the comparison was farther than the standard (figure 11), the opposite occurred: subjects required more disparity in the comparison with match the standard than would be predicted with perfect constancy. Both these behaviours are consistent with the hypothesis that subjects underestimated the change in distance between the comparison and standard.

It is important to note that subjects achieved quite accurate constancy in the condition in which the comparison and standard stimuli had the same spatial frequency in the world. Those particular conditions are indicated by the large circles in the two figures. It seems that people are better able to appreciate the same depth in a stimulus of fixed relief viewed from different distances than in stimuli in which the scale of the relief differs. This makes sense, because a person could learn the relationship between the disparity fields created by the same relief viewed from different distances.

There were some consistent individual differences. Two subjects required more disparity with increasing spatial frequency, one required less and one required the essentially same disparity. Those behaviours were observed in these individuals both when the comparison was nearer and farther than the standard. We did not observe such individual differences in the effect of spatial frequency in experiment 1, so we wondered why they occurred in experiment 2. The most likely cause is an important difference in the way the stimuli were rendered in the two experiments. In experiment 1, vertical disparity was zero throughout the images. This was acceptable in that experiment, because we did not manipulate the stimulated viewing distance and errors in estimating viewing distance from incorrectly rendered vertical disparity affected the reference and comparison equally. Experiment 2 involved changes in simulated distance, so we rendered non-zero vertical disparities as appropriate for each simulated viewing distance. The perspective projection used to render the appropriate disparities caused the slants of the sides of each triangular ridge to differ notably as a function of elevation. Specifically, a ridge at the centre of the stimulus appeared to have similar slants for the upper and lower parts of the triangle while a ridge at the top of the stimulus yielded notably different slants for the upper and lower parts (the slant being less for the lower part and greater for the upper). This became most notable as spatial frequency increased. Perhaps some subjects perceived more depth in the corrugation waveform when this change in slant became quite notable, so they accepted less disparity in a high-frequency comparison to yield the same apparent depth as a standard of lower frequency. Perhaps other subjects were unaffected by this perspective effect. If that were the case, it explains why individual differences were observed in experiment 2, but not experiment 1.

**Figure 12.**
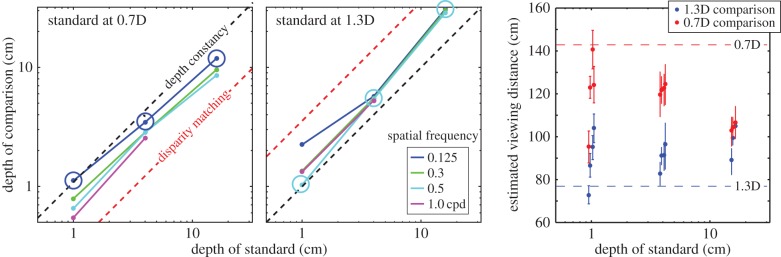
Matching depths for different viewing distances and spatial frequencies. The depth variation in the comparison stimulus that was perceived as the same as the variation in the standard stimulus is plotted for different amounts of depth variation in the standard and different spatial frequencies in the comparison. The depth variations are the distance from peak to trough in cm. Data for different frequencies are represented by different colours. The left panel shows the matches when the distance of the standard was 0.7D and that of the comparison was 1.3D. The middle panel shows the matches when the distances of the standard and comparison were 1.3 and 0.7D, respectively. The dashed diagonal lines indicate depth constancy. The circles indicate the conditions in which the spatial frequency of the comparison in cycles per cm was the same as that of the standard. The right panel shows equivalent distance for different distances, spatial frequencies and corrugation depths. Equivalent distance is the distance of the comparison for which the disparity settings would be veridical if the only error is in the estimate of viewing distance. The blue line represents the viewing distance of the comparison when it was at 1.3D and therefore nearer than the standard, and the red line represents the distance of the comparison when it was at 0.7D and therefore farther than the standard. The blue and red dots represent the data for the near and far comparisons, respectively (different dots representing the data for different spatial frequencies).

In figure 12, we re-plotted the data from figures 10 and 11 to make it easier to compare observers' responses to the expectation for depth constancy. The figure plots the peak-to-trough depth of the comparison in cm as a function of the depth of the standard also in cm. The left and middle panels show those data when the comparison was respectively nearer and farther than the standard. The black dashed lines represent depth constancy, i.e. where the depth variations in the comparison and standard corrugations are identical. The red dashed lines represent disparity matching, i.e. where the disparities created by the standard and comparison are the same. The coloured lines and circles represent the pooled data. When the standard stimulus was at 0.7D and the comparison at 1.3D, the depth variation in the comparison stimulus was somewhat overestimated relative to the standard. Thus, subjects perceived the two as having the same depth when the comparison had slightly less depth than the standard. The opposite occurred when the standard stimulus was at 1.3D and the comparison at 0.7D; here, the depth of the comparison stimuli at 0.7D was underestimated relative to the standard, so subjects perceived the two as having the same depth when the comparison had slightly more depth than the standard.

The spatial frequency of the standard at the retina was always 0.3 cpd. The spatial frequencies of the comparison that correspond to the same relief in the world are 0.16 cpd when the standard and comparison are respectively at 0.7 and 1.3D, and 0.56 cpd when the standard and comparison are respectively at 1.3 and 0.7D. These stimuli are indicated by the large circles in the figure. The settings in both cases are generally closer to depth constancy than the settings for other spatial frequencies, which again shows that people are more constant in judging the depth of fixed relief across different distances.

Clearly, the data were better described by the depth–constancy prediction than by disparity matching, but it is worth noting that the deviations from constancy were nearly all in the direction of disparity matching. We can quantify the degree to which observers compensated for viewing distance by calculating the *equivalent distance* associated with each data point. The equivalent distance is the distance estimate that would have produced the data under the assumption that the only error is in the distance estimate [23]. The blue and red horizontal lines represent the viewing distance of the comparison when it was respectively at 1.3 and 0.7D. The blue and red dots represent the data for those two situations, different dots representing data from different spatial frequencies. There was a fairly consistent error for all conditions, again in the direction of not fully compensating for the change in distance between the comparison and standard. In addition, the error increased when the depth of the standard was large, because, in that case, it approached the disparity-gradient limit and its apparent depth decreased.

## 4. Discussion

### (a) Comparison of previous and current observations

We found that people can compensate for spatial-frequency-dependent differences in stereoscopic thresholds and the disparity-gradient limit, provided that the disparity variation is large enough to exceed the threshold limit yet small enough to not encroach on the gradient limit. The range of disparities between those limits is large at low spatial frequencies (e.g. from 0.06 to 0.25 cpd, the range is roughly 500-fold) but becomes vanishingly small at high frequencies (e.g. at 2 cpd, it is approx. 10-fold). Additionally, we found that people are able to compensate reasonably well for changes in viewing distance and thereby achieve nearly complete depth constancy. Unlike previous experiments, our stimuli presented vergence, focus cues and vertical disparity signals that were appropriate for the changes in viewing distance. We examined whether the inclusion of the three affected the amount of constancy by comparing our results with all signals correct to previous results in which only one or two were correct.

From among several studies that examined stereoscopic depth constancy with changes in object distance [21,22,24,26,32], we selected four that employed similar stimuli and techniques [23,25,33,34]. From the data in each study, we calculated equivalent distance as a function of simulated distance. We then found the model that best fit the data via linear regression. The slope of the regression line was the estimate of the amount of observed constancy. If the slope was 1, constancy was perfect. If it was 0, no compensation for distance was observed. Table 1 shows these slopes for the four studies. The column labels refer to the signals that were appropriate for the viewing distance: V for vergence, VD for vertical disparity and F for focus cues (blur and accommodation). Note that only one study—Watt *et al*. [23]—presented all three signals correctly. Although there are too few entries to be definitive, it appears that providing vergence alone or vertical disparity alone yields rather incomplete constancy. Providing two or three signals seems to increase constancy considerably.

**Table 1.** Constancy gains in previous studies.

study	V	VD	V and F	V and VD	V, F and VD
Bradshaw^a^	0.19	0.16	x	0.36	X
Cumming^b^	0.17	0	0.40	x	x
Johnston^c^	x	x	0.26	x	x
Watt^d^	x	x	x	0.35	0.60

^a^From fig. 5*a*; 80 cm patch; averaged across subjects.

^b^From fig. 2*a,b*; averaged across subjects.

^c^From fig. 7; averaged across cylinder depths and subjects.

^d^From fig. 14; averaged across subjects.

Table 2 shows the gains we observed in experiment 2. The rows are different depths in the standard stimulus, and the columns are the slopes from all the data or only from the data in which the standard and comparison stimuli had the same spatial frequency in the world. Stimuli with the same spatial scale in the world yielded greater depth constancy (0.63 on average) than all of the stimuli combined (0.37). The amount of constancy we observed with correct vergence, vertical disparity and focus cues is generally greater than the amount observed in the previous studies, which presented only one or two signals correctly. The constancy we observed is similar to that reported by Watt and co-workers when all three signals are correct.

**Table 2.** Constancy gains in experiment 2.

depth (cm)	all data	same relief
1	0.52	1.03
4	0.48	0.61
16	0.11	0.24
mean	0.37	0.63

It is notable that we observed systematically greater constancy when the depth interval was small (1 cm) than when it was large (16 cm). Over a 16-fold range in depth, we observed an approximately fourfold reduction in constancy. Johnston [34] observed a similar effect. Over a threefold range in depth, she observed an approximately 1.5-fold reduction in constancy. The reduction in constancy with large depth intervals is probably owing to encroachment of the disparity-gradient limit.

We also observed consistently greater constancy when the depth variation had the same spatial frequency in the world rather than the same retinal frequency. Collett *et al*. [21] observed a similar effect. It is also interesting to note that Burbeck [35] observed a similar effect in the luminance domain: spatial-frequency discrimination was better when the two stimuli had the same frequency in the world, but different retinal frequencies, as opposed to when they had the same retinal frequency, but different world frequencies.

### (b) Determining perceived depth with different psychophysical procedures

The upper-disparity limit and the lower-disparity thresholds lead to an interesting problem in interpreting the psychophysical data. The problem is illustrated by Brady & Field [36], who examined perceived contrast in the luminance domain. Using the method of adjustment they compared the perceived luminance contrast of standard and comparison stimuli at different spatial frequencies. Near threshold, the same grating was perceived on some trials and was not perceived on others. When the grating was not perceived, the subjects were instructed to set the contrast of the standard stimulus to zero. When the grating was perceived, subjects set the standard to the same contrast as the comparison and thus exhibited nearly perfect constancy. Imagine the above was repeated for 20 trials and that the comparison was not perceived on half the trials and was perceived on the other half. Also, imagine that the subjects exhibited perfect contrast constancy when they perceived the comparison stimulus. Given these assumptions, subjects would set the standard contrast to zero on 10 trials and to the same contrast as the comparison on 10 trials. The data would fall into two categories and Brady and Field argued that this is what occurred in their experiment. Now, imagine the analogous experiment using a 2-alternative, forced-choice procedure (2AFC) such as the one in our experiments. On the trials in which the stimulus was not perceived, the standard would be selected every time. On the other trials in which the comparison was perceived, subjects would select the comparison half the time and the standard the other half. Combining the two scenarios, the comparison would be selected as having more perceived contrast on 25% of the trials, so the data would suggest that the comparison had less perceived contrast even though it had the same perceived contrast on half the trials. Thus, the 2AFC method is unable to distinguish between the two-state scenario described by Brady and Fields and a one-state scenario in which the comparison has less perceived contrast. The same reasoning could be applied to our data on stereoscopic depth constancy (i.e. figures 4 and 11).

To examine this possibility, we repeated experiment 1 using the method of adjustment with two of the four subjects (one experienced and one naive). The disparity of the standard stimulus was 0.25, 0.5, 1, 4 or 16 arcmin. Subjects adjusted the disparity of the comparison until the two had equal perceived depth. When subjects could not match the depth of the comparison with the standard, they were told to skip the trial. Conditions were repeated 24–40 times for each combination of spatial frequency and disparity amplitude (SF = 0.0625, 0.125, 0.5 and 1 cpd; disparity = 0.25, 0.5, 1, 4 and 16 arcmin). The experienced observer was able to complete the task on at least 87% of trials across all conditions. The naive observer was able to complete the task on at least 96% of trials when disparity was greater than 1 arcmin, at least 75% of trials when disparity was 0.5 arcmin, and 43–67% of trials when disparity was 0.25 arcmin. When we include only the successfully completed trials, the disparities for equal perceived depth near detection threshold remained bandpass (see electronic supplementary material). For amplitudes well above detection threshold, the data flattened and exhibited depth constancy. Thus, our data show convincingly that the effect of spatial frequency depends strongly on whether the disparity amplitude is near threshold or above threshold and is not an artefact of using a 2AFC procedure.

### (c) Disparity manipulations for compression and discomfort reduction

There have been many reports of viewer discomfort associated with viewing stereoscopic displays [37]. One cause of the discomfort is the conflict between vergence distance and accommodative distance [38,39]. Many researchers have proposed methods for adjusting the disparities in the content in order to reduce the magnitude of the vergence–accommodation conflict and thereby reduce discomfort [40,41]. There have also been several proposals for compression algorithms for stereo content [41–43]. Like compression algorithms for non-stereo content, the stereo algorithms aim to incorporate properties of human vision to preserve perceptible features while discarding imperceptible ones. Our results are relevant to the development and evaluation of disparity-remapping and compression algorithms, because the results can inform us about the kinds of disparity adjustments that will or will not cause notable distortions in perceived depth.

**Figure 13.**
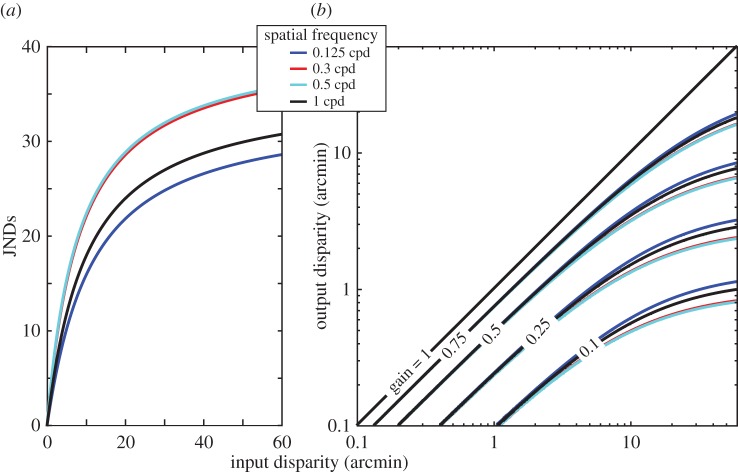
Disparity manipulation in the algorithm of Didyk *et al*. [41]. (*a*) Transducer functions from Didyk *et al*. used to linearize perceived depth from disparity. JNDs are plotted as a function of input disparity for four spatial frequencies. The functions are compressive, because JNDs become larger with increasing disparity, so a given change in large disparity creates a smaller range of JNDs than the same change in small disparity. (*b*) The input–output mapping functions from Didyk *et al*. The labels to the sets of curves are the reductions in JNDs of possible re-mappings from 1 (no change in disparity) to 0.1 (significant reduction). The colours of the curves indicate the functions for four different spatial frequencies.

Didyk *et al*. [41] described a perceptually based method for disparity re-mapping. The method uses JNDs in disparity to guide re-mapping. The idea is to do disparity adjustments in a perceptually linear space as is often done in the luminance domain. Disparities are adjusted in units of JNDs, the JNDs being derived from psychophysical experiments conducted by Didyk and co-workers. Those experiments yielded disparity-discrimination thresholds across a range of corrugation frequencies and disparity amplitudes. Figure 13*a* shows the transducer functions derived from their data. These functions map input disparities at different frequencies into JNDs. One then adjusts disparities, frequency by frequency, by equivalent amounts in JND units in order to determine the output disparities. The mapping from input disparity to output disparity is shown in figure 13*b*. Mapping for gains of 1 (i.e. no adjustment) to 0.1 (significant adjustment) are represented by the various curves, different colours for different corrugation frequencies.

A potential problem with the approach of Didyk *et al*. [41] is that discrimination data do not necessarily predict appearance. This point is exemplified by the phenomenon of contrast constancy in the luminance domain. Constancy in that domain occurs over a wide range of spatial frequencies provided that the stimuli are suprathreshold [14]. For example, the apparent contrasts of a high-frequency grating (say 20 cpd) and a medium-frequency grating (5 cpd) are the same when the contrasts are physically the same, provided that the stimuli are above threshold. This phenomenon is not predicted by contrast-discrimination data, because the JNDs for higher frequencies are larger than for medium frequencies [44]. Our data show that human observers are depth constant—i.e. they perceive the same depth variation across a wide range of corrugation frequencies when the disparities are physically the same—provided that the disparity is sufficiently above detection threshold. From figures 4 and 5, one can see that depth constancy occurs for disparities greater than 1–4 arcmin. That means that the perceived relief of a surface containing suprathreshold disparities at multiple frequencies should appear undistorted if the disparities in different frequency bands are not altered. At disparities smaller than 1–4 arcmin, the perceived relief would be closest to the actual relief if detection thresholds were taken into account (i.e. amplify disparities at low and high corrugation frequencies relative to disparities at 0.3–0.5 cpd). The algorithm of Didyk and co-workers behaves in a fashion that is reasonably compatible with our data. When the gain of the disparity adjustment is 0.75–1, their algorithm preserves the ratio of disparities across corrugation frequency for input disparities of 10 arcmin and less. From our data, such adjustments should lead to acceptable appearance although the overall attenuation of disparity would yield a flattened percept relative to the original content. When the gain of the adjustment is 0.5 or lower, the algorithm alters the ratios of disparities across frequency; specifically, it attenuates disparities at medium frequencies more than at low and high frequencies. The attenuation differences across frequency are, however, small, so appearance should not be significantly altered apart from the overall flattening. A departure from what our data would suggest occurs when the input disparities are small (roughly less than 1 arcmin). Here, the algorithm maps disparities of low and high corrugation frequencies to the same value as medium frequencies. From our data, one would amplify the disparities of low and high frequencies in such cases.

### (d) Misperception of depth when interpupillary distance is misestimated

An important part of the capture–presentation pipeline for generating three-dimensional percepts is the assumed IPD [45,46]. Stereoscopic content for wide distribution (e.g. cinema, television) must assume an IPD for the intended audience. Here we ask how errors in the assumed IPD are likely to affect three-dimensional percepts. That is, what is likely to happen when the viewer's IPD differs from the one used in generating the content? Note that this discussion is about the generation of stereoscopic content and the assumption the content creators can reasonably make. It is not about how the viewer's visual system compensates for changes in viewing distance, which we investigated in experiment 2.

**Figure 14.**
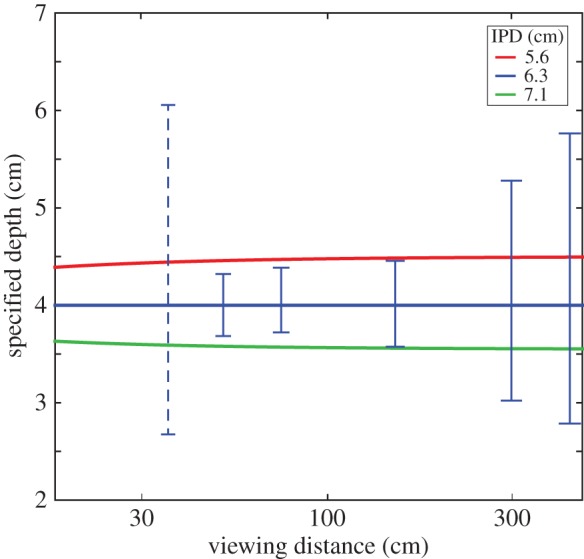
Depth from disparities for viewers with different interpupillary distances (IPDs). Depth from disparity is plotted for different viewing distances and for viewers with different IPDs. The red, blue and green curves represent the depths for viewers with IPDs corresponding respectively to the fifth, 50th and 95th percentiles of the adult population. The blue vertical lines represent the just-notable differences converted into units of depth from experiment 1.

To examine how errors in assumed IPD are likely to affect percepts, we first calculated how an accurate mapping from disparity to depth is affected by the viewer's IPD. From Dodgson [47], we determined the IPDs that correspond to the fifth, 50th and 95th percentiles in the adult population. These are respectively 5.6, 6.3 and 7.1 cm. We calculated for an observer with the average IPD of 6.3 cm, the disparities created by a 4 cm ridge on an otherwise frontal plane at different distances. We then calculated the specified depth if those disparities were presented to observers with IPDs of 5.6 and 7.1 cm. The red and green curves in figure 14 correspond to those depths when the stimulus is viewed at different viewing distances. We next converted the smallest JNDs in disparity from experiment 1 (figure 7) into units of depth (spatial frequency = 0.3 cpd). The blue vertical lines in the figure represent those depth JNDs; they are the estimates of the depth variations that would be just discriminable. The dashed line represents JNDs when the base disparity was 64 arcmin where the disparity-gradient limit clearly affected the data. The range of depth variations we calculated from those JNDs increases with viewing distance. The important point is that the JND range is not generally smaller than the range for people with different IPDs. We conclude that assuming a population average for IPD will generally not produce notable differences in perceived depth for most individuals.

## 5. Conclusion

In experiment 1, we found that depth constancy occurs across a broad range of corrugation frequencies provided that the disparity amplitude is not close to the lower-disparity threshold or the upper-disparity limit. Low spatial frequencies avoid the disparity-gradient limit at all but the largest amplitudes and therefore can convey more apparent depth than other frequencies. In experiment 2, we found that depth constancy is reasonably accurate across changes in viewing distance when vergence, vertical disparity and accommodation provide appropriate distance information. Interestingly, constancy was most accurate when the relief had the same spatial frequency in the world and not the same spatial frequency on the retina.

## Supplementary Material

Method of Adjustment Data

## Data accessibility

The data are accessible through the laboratory at http://bankslab.berkeley.edu/projects.
