# Supplementary material for: Stereoscopic depth constancy
Source: Philos Trans R Soc Lond B Biol Sci. 2016 Jun 19;371(1697):20150253. doi: 10.1098/rstb.2015.0253 (PMC4901447; doi:10.1098/rstb.2015.0253)
Supplement: Method of Adjustment Data [file rstb20150253supp1.pdf]

Guan P and Banks MS 2016. Stereoscopic depth constancy. *Phil. Trans. R. Soc. B.* **371** doi: 10.1098/rstb.2015.0253

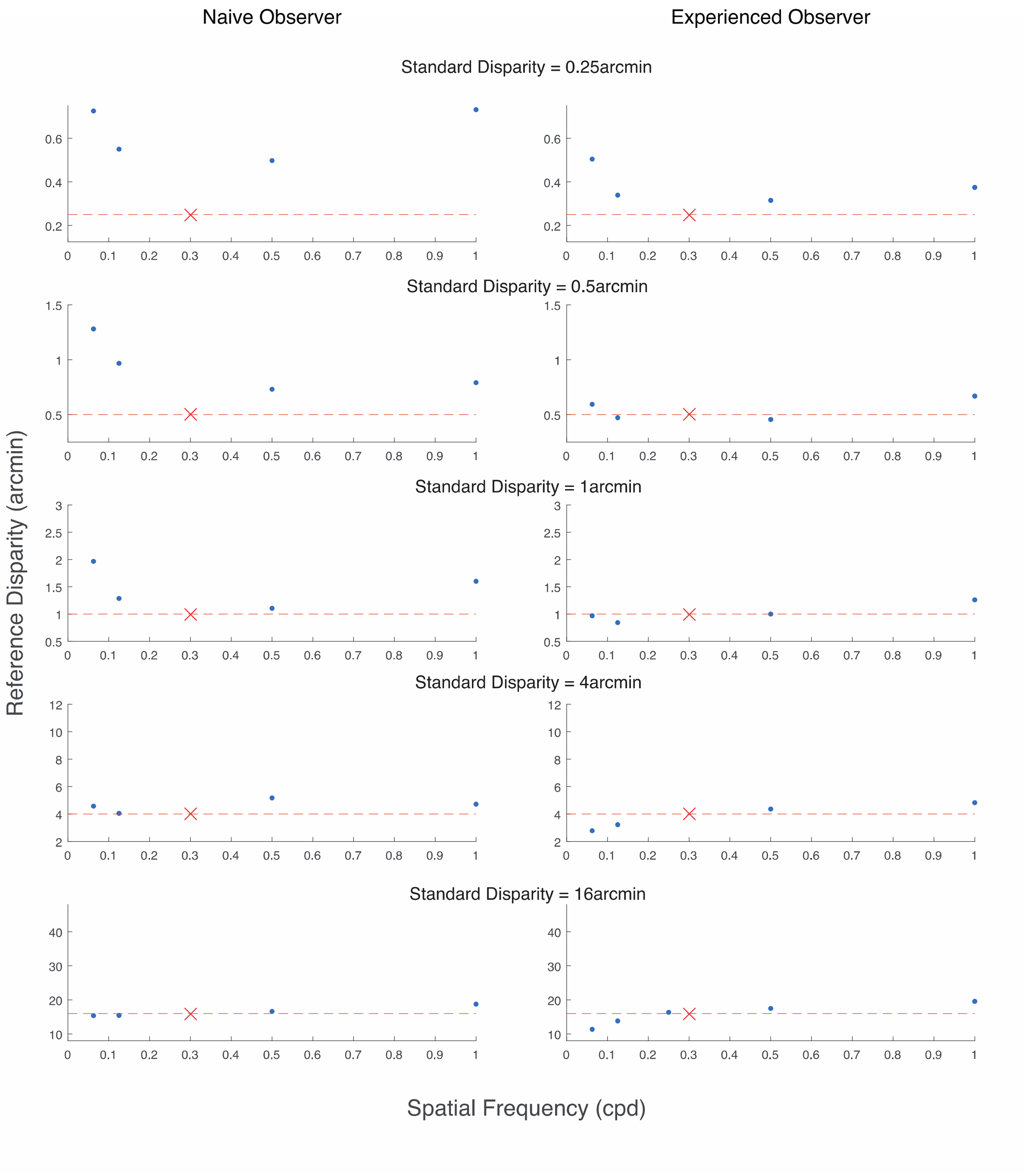

Supplementary Figure 1. Perceived depth for naïve and experienced observer using method of adjustment. Data for the naïve and experienced observers are presented on the left and right respectively. The red X shows the disparity and spatial frequency of the reference and the blue dots represent the average disparities of the completed reference trials (i.e. trials that were not skipped) obtained using method of adjustment. The experienced observer exhibits bandpass behavior at the smallest reference disparity of 0.25arcmin and depth constancy at disparities larger than 0.25arcmin. The naïve observer exhibits bandpass behavior for disparities up to 1arcmin and depth constancy for disparities greater than 1arcmin. The naïve observer completed at least 43% of trials at 0.25arcmin, 75% of trials at 0.5arcmin, and 96% of all other trials. The experienced observer was able to complete at least 87% of trials across all conditions.
